# Loading Rate and Temperature Interaction Effects on the Mode I Fracture Response of a Ductile Polyurethane Adhesive Used in the Automotive Industry

**DOI:** 10.3390/ma15248948

**Published:** 2022-12-14

**Authors:** Mael Perez, Alireza Akhavan-Safar, Ricardo J. C. Carbas, Eduardo A. S. Marques, Sabine Wenig, Lucas F. M. da Silva

**Affiliations:** 1 Departamento de Engenharia Mecanica, Faculdade de Engenharia da Universidade do Porto, 4200-465 Porto, Portugal; 2 Institute of Science and Innovation in Mechanical and Industrial Engineering (INEGI), 4200-465 Porto, Portugal; 3 Sika Automotive AG, Kreuzlingerstrasse 35, 8590 Romanshorn, Switzerland

**Keywords:** adhesive joints, loading rate, fracture energy, polyurethane, temperature

## Abstract

Due to their high elongation at failure and damping capacity, polyurethanes are one of the main types of adhesives used in automotive structures. However, despite the wide range of applications of adhesives, their fracture mechanics behavior is still poorly studied in the literature, especially when both the loading rate and ambient temperature change. Accordingly, the main aim of the current work is to deal with the research gap. In the current research, mode I fracture energy of a ductile polyurethane adhesive with adaptive properties for its industrial application is determined at different test speeds and temperatures. Tests were done at quasi-static, intermediate, and high-speed levels and each at three different temperatures, including low, high, and room temperature. Mode I fracture toughness was determined using DCB tests. Increasing the loading rate from quasi-static to 6000 mm/min was found to significantly increase the maximum strength of the tested DCBs (from 1770 N to about 4180 N). The greatest sensitivity to the loading rate was observed for the DCBs tested at room temperature, where the fracture energy increased by a factor of 3.5 from quasi-static (0.2 mm/min) to a high loading rate (6000 mm/min). The stiffness analysis of the DCB samples showed that the transition from below the *T_g_* to room temperature decreases the bond stiffness by about 60%, while a further temperature increase (from 23 °C to 60 °C) has no significant effect on this parameter. Since polyurethane-bonded joints often experience a wide range of temperatures and loading rates in service, the obtained results can be used to design these joints more securely against such loading/environmental conditions.

## 1. Introduction

Some of the most common methods used for material joining, such as riveting or welding, still have significant practical issues. For example, welding dissimilar materials is very complex, and riveting adds stress concentrations because of the holes made in the material. Within this context, adhesive bonding has gained a lot of popularity recently: it is not affected by the issues these other methods have, and allows for lighter structures while having good fatigue resistance thanks to uniform stress distribution present within the joint [1]. It is also seen as one of the best ways to join composite structures and highly dissimilar materials. Furthermore, an increased awareness to global climate change has made reducing structural weight a key topic in the automotive industry, as it leads to a significant reduction in the energy consumption of vehicles. The same goes for many other industries, such as those operating in the aerospace or naval sectors. Adhesively bonded structures are thus being extensively used to answer the different requirements of these industries. However, designing complex structures with modern structural adhesives is still a challenge, compounded by the fact that the joints need to resist complex loading conditions under a wide range of environmental conditions.

In the automotive industry, for example, temperatures can vary greatly during the operation of a vehicle and the bonded parts can be subjected to high loading rates under impact condition. Therefore, it has become highly important to study the performance of bonded joints under these different conditions in order to ensure the safety of the occupants and ensure the smooth and long-lasting operation of the vehicle. To design efficient adhesively bonded joints for use in automotive construction, the performance of the bonded materials must be characterized under different loading rates and temperatures, as the viscoelastic and viscoplastic behaviors of the adhesive are highly dependent on these parameters.

The effect of several parameters, such as the presence of additives [2,3], cyclic loading, joint geometry [4], etc., on the fracture energy of bonded joints have been studied in the literature. However, experimental studies on the effect of loading rate on the fracture energy are still rare and the dynamic fracture behavior of adhesives is a relatively new field of study, especially when the influence of both the loading rate and the temperature are taken into account. However, a few authors have already studied the influence of the loading rate [5,6,7] on the impact strength of bonded joints [8,9,10], almost always using epoxy-based adhesives.

For epoxy adhesives, the fracture energy in mode I has been shown to decrease as a function of the loading rate by Bascom et al. [11], Bitner et al. [12], Hunston et al. [13], Lataillade et al. [14], Blackman et al. [15,16], Raghavan et al. [17], and Karac et al. [18]. Increasing the loading rate can lead to a decrease in the fracture energy of epoxies. Increasing the loading rate causes a decrease in the elongation at failure while typically leading to an increase in the strength of the adhesive. Consequently, the fracture energy as a product of the displacement and load can be reduced or increased. However, with epoxy adhesives, the increase in strength usually cannot compensate for the decrease in displacement, resulting in a reduction in fracture energies. It should be noted that additives contained in the epoxies can change the behavior of the adhesives, leading to an increase in the fracture energy by increasing the loading rate [11]. There are also studies where the authors show that during a single test the loading rate changes as the crack size grows [19]. For the loading rate, a possible explanation was given by Raghavan et al. [17], saying that a higher loading rate decreases the viscoelastic deformation ahead of the crack-tip, which reduces the fracture energy. May et al. [20] proposed a different justification, stating that after a certain value of crack propagation is reached, the conditions ahead of the crack-tip change from isothermal to adiabatic, thus heating the adhesive and diminishing its local properties. However, there are also reports of increases of fracture energy caused by an increase in loading rate. Such reports can be found in the works of Kinloch et al. [21], Biel et al. [22], Carlberger et al. [23], Marzi et al. [24], Borges et al. [25], May et al. [20], and Nunes [19]. It should be noted, however, that the loading rate dependency of these adhesives also implies a precise determination of the true loading rate along the bondline, which varies drastically from the nominal/engineering loading rate. Indeed, a constant crosshead displacement rate does not necessarily generate a constant effective loading rate as shown by Nunes et al. [26]. The use of SHPB specimen over DCB or inversely does not seem to matter, as the mode I fracture energy obtained with either of these types of specimens has been found to lead to similar results [27].

Regarding the temperature dependences, Kumpenza et al. studied the effect of temperature on the tensile behavior of different adhesive systems [28]. Banea et al. [29] studied an adhesive at room and high temperature, showing a sizeable decrease of the fracture energy of mode I for temperatures above the *T_g_*, but a relatively stable behavior when below the *T_g_*. The explanation for the results below the *T_g_* of the adhesive is the fact that while the increase in temperature reduces the strength, it also increases the ductility which adds plastic deformation ahead of the crack-tip. Both these changes can increase or decrease the fracture toughness. This temperature-sensitive behaviour, which reduces the adhesive’s property above the *T_g_*, was also studied by Banea et al. [30,31], Jia et al. [32], and Bernasconi et al. [33]. 

Compared to epoxies, studies on the mechanical characterization of polyurethane as a function of loading rate and temperature are rare, while nowadays these types of adhesives are widely used in various industrial sectors. Tang et al. [34,35] investigated the effects of additives on the thermal characteristics and flammability of polyurethane. Jia et al. [36] analyzed mode I fracture toughness of a ductile polyurethane adhesive at different loading rates limited up to 500 mm/min and at different low temperatures all below the *T_g_* of the adhesive. Their work also shows that the increase in loading rate (or a decrease in temperature) decreases the fracture energy. However, at lower temperatures, the decrease linked to the loading rate is less important. 

As mentioned above, although extensive work has been carried out on epoxies, similar studies on joints bonded with ductile polyurethanes adhesives, taking into account both the loading rate and temperature, are very rare. The work performed in the previous studies are limited to low loading rates and to the temperatures below the *T_g_* of the adhesive. 

Accordingly, the current study provides experimental data on the fracture behavior of a special purpose ductile polyurethane adhesive as a function of loading rate from quasi static conditions (0.2 mm/min) to high loading rate (6000 mm/min) and at various temperatures below and above the *T_g_* of the adhesive.

## 2. Experimental

### 2.1. Material

A polyurethane-based adhesive was used in this study. This is a ductile adhesive with mechanical properties adapted for industrial applications. The *T_g_* of the tested adhesive is −5 °C. The cure cycle of the adhesive is composed of a stage of 24 h at room temperature followed by with an additional 4 h at 80 °C, during a post-curing process. The substrates used for the DCB joints are made of a high strength steel (PM300). To prepare the surface of substrates, they were sand-blasted and then cleaned with acetone before the adhesive application. Table 1 shows the physical and mechanical properties of the adhesive at room temperature, with the tensile properties resulting from quasi-static testing of dogbone shaped bulk specimens. Physical properties were provided by the adhesive manufacturer. 

### 2.2. Geometry and Manufacturing Procedure

**Figure 1:**
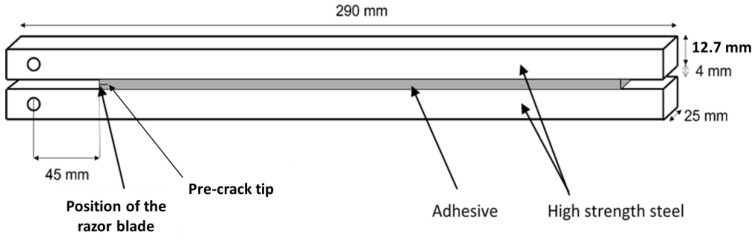
Geometry of the DCB specimen, dimensions in mm.

Figure 1 shows the geometry of the tested joints.

The adhesive thickness used in the DCB joints under analysis was 4 mm. This specific value was chosen to be consistent with the thickness found on the typical applications of this adhesive in car bodies. Furthermore, this adhesive is also suitable for use in wind blades construction, where the bondlines often exceed 4mm. This thickness was also chosen knowing that the joint’s properties are a function of the adhesive thickness [4,37,38,39].

The manufacturing process of the DCB joints started by sand-blasting the surfaces of both specimens. The sand-blasting procedure removes the iron oxides and creates a surface which is better suited for adhesion. The surfaces are then degreased with acetone. To ensure the presence of an initial crack, a sharp razor blade was introduced at the mid-thickness of the 4 mm bondline. To ensure this dimension, the razor blade is placed between two steel spacers bearing a thickness slightly below 2 mm and with its position adjusted to obtain an initial crack length at 45 mm. On the other side of the joint, a 4 mm thick steel spacer was placed to help control the bondline thickness. 

A common mold used for DCB manufacturing usually consists of top and bottom plates with holes for inserting guide pins that hold the DCBs in place during the manufacturing process. However, due to the very low viscosity of the adhesive used in this study and on the other hand the very thick bondline (4 mm) needed it was necessary to modify the regular mold by adding additional 3D printed parts to make sure that a sufficient amount of the poured adhesive remains within the joint during the curing process. Accordingly, the used mold was called special.

To avoid adhesion to the supporting tools, release agent was applied to the spacers, the blade, and the 3D-printed parts. All blades were removed before testing the joints.

**Figure 2:**
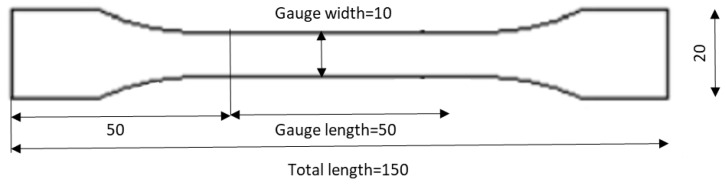
Geometry of the bulk tests specimen (dogbone) according to BS 2782 with the thickness of 2 mm, not shown in this image, (dimensions in mm).

For manufacturing tensile specimens, the French NF T 76-142 standard was followed. Accordingly, adhesive plates with a thickness of 2 mm were produced in a special steel mold containing a silicone rubber frame which provides hydrostatic pressure under compression and minimizes the appearance of voids. The adhesive is applied to this rubber frame, the mold is closed, and it is then introduced in a hot-plate press. The cure proceeds under a well-controlled amount of pressure and temperature for 24 h to ensure that the curing process of the adhesive goes well. As was the case for the DCBs, bulk plates were also subjected to a high temperature post curing process. The post-curing cycle is divided into 3 phases: the heating ramp, a stage at 80 °C that lasts for 4 h, and the cooling process. Figure 2 shows the geometry of the dogbone samples tested under tensile loading.

### 2.3. Test Procedures

Different loading rates were considered to conduct mode I fracture tests, including a quasi-static rate at 0.2 mm/min, an intermediate rate at 200 mm/min, and a high rate at 6000 mm/min. For each loading rate, the joints were tested at different test temperatures including low temperature at −30 °C (LT), room temperature at 23 °C (RT), and high temperature at 60 °C (HT), always under mode I loading conditions. Test conditions considered in this study are summarized in Table 2. For the DCB tests, the quasi-static, intermediate speed, and high-speed tests were conducted using a hydraulic test machine (INSTRON^®^ 8801) equipped with a load cell of 100 kN where the sampling rate was set to 10 Hz and 10 kHz for the quasi static and high loading rate, respectively. The load and displacement recorded by the machine were used to analyze the results.

A specially designed chamber was used for high and low-temperature tests, allowing to keep the temperature of the sample constant during the test. The samples were kept in the chamber for 10 min at the required temperature to ensure that the adhesive layer fully reached the target temperature before testing. Adhesive temperature was also monitored during the test using a thermocouple attached to the bondline.

## 3. Data Reduction Approach

The fracture energy was obtained using the Compliance Based Beam Method (CBBM), a data-reduction scheme which does not require the exact crack length to measured but instead determines an equivalent crack length, calculated from the compliance of the test specimen. Accordingly, the mode I critical fracture energy is given by [40]:

(1) $$G_{IC}=\frac{6P^{2}}{b^{2}h}\left(\frac{2a_{eq}^{2}}{h²E_{f}}+\frac{1}{5G}\right)$$

where *a_eq_* is an equivalent crack length obtained from the experimental compliance and accounting for the fracture process zone at the crack tip, *h* is the thickness of the substrates, *b* is the specimen width, *P* is the load, *E_f_* is a corrected flexural modulus to account for all phenomena affecting the load-displacement curve, such as stress concentrations at the crack tip and stiffness variability between specimens, *G_IC_* is the mode I fracture energy, and *G* corresponds to the shear modulus of the adherents. The *E_f_* is obtained to include the effect of the added stress concentration around the crack tip which impacts the initial linear part of the load-displacement curve, as well as the rotation of the substrate [41]:

(2) $$E_{f}={\left(C_{0}-\frac{12\left(a_{0}+\left|\Delta \right|\right)}{5Gbh}+\frac{1}{5G}\right)}^{-1}\frac{8{\left(a_{0}+\left|\Delta \right|\right)}^{3}}{bh^{3}}$$

with *a*_0_ and *C*_0_ being, respectively, the initial crack length and the initial compliance. Δ is a correction factor for the crack length given by [41]:

(3) $$\Delta =h\sqrt{\frac{E}{11G}\left(3-2{\left(\frac{\Gamma }{1+\Gamma }\right)}^{2}\right)}$$

where

(4) $$\Gamma =\frac{1.18E}{G}$$

where *E* is the Young’s Modulus of the substrate.

The *a_eq_* is obtained with Timoshenko’s beam theory which gives this equation [41], with *C* being the specimen’s compliance:

(5) $$C=\frac{\delta }{P}=\frac{8a_{eq}^{3}}{bh^{3}E_{f}}+\frac{12a_{eq}}{5Gbh}$$

The crack length correction Δ can be determined using a linear regression based on three different results, at different loading rates.

## 4. Results and Discussions

**Figure 3:**
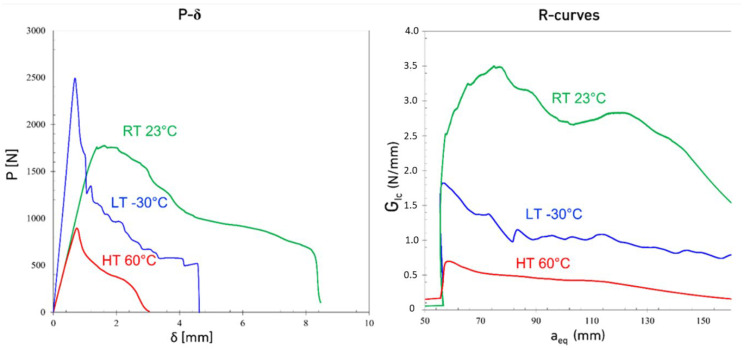
Representative Load-displacement and R-curves of DCB joints under quasi-static loading rates (0.2 mm/min) at different temperatures.

**Figure 4:**
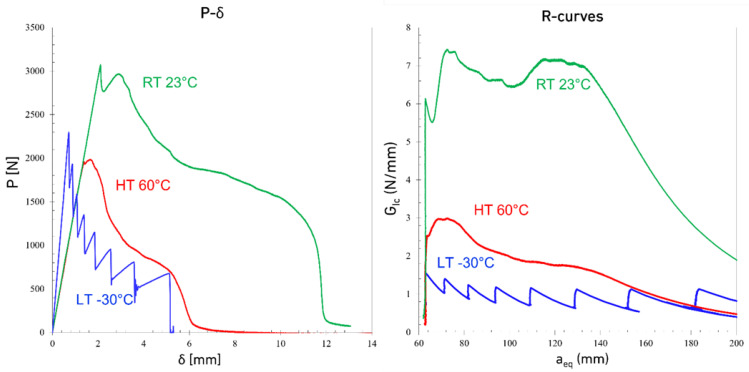
Representative Load-displacement and R-curves of DCB joints under speed loading rates (200 mm/min) at different temperatures.

**Figure 5:**
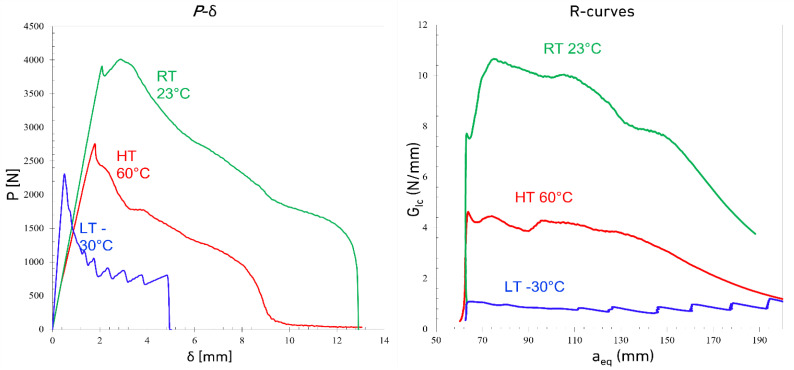
Representative Load-displacement curves of DCB joints under high loading rates (6000 mm/min) at different temperatures.

Figure 3, Figure 4 and Figure 5 show the typical load displacement and the corresponding R-curves of the DCBs tested at different loading rates and temperatures. The load-displacement curves show two different types of behavior: a brittle one, with higher Young’s modulus for LT conditions and a more ductile response for the tests carried out above the *T_g_* of the adhesive (considering a *T_g_* of −5 °C). Both the temperature and the loading rate noticeably change the response of the adhesive. As shown in Figure 3, Figure 4 and Figure 5, by increasing the loading rate from a quasi-static rate to the higher test speeds for joints tested at LTs, crack propagation becomes more gradual and a saw-like response is observed. Increasing the loading rate at LTs made the adhesive more brittle and consequently, stepwise crack propagation was observed as a common failure mode in DCB joints with brittle adhesives, as shown in Figure 4 and Figure 5. In this type of failure, the energy stores in a small area at the crack tip (due to the low ductility of the adhesive) and is released when the energy reaches a critical value. The energy release process occurs through sudden crack propagation. The length of crack propagation at each step is defined by the amount of energy stored at the crack tip. The work done in crack propagation is similar to this energy.

It is also shown in Figure 3, Figure 4 and Figure 5 that while the LT *G_Ic_* is above the high-temperature results at quasi-static loading rate, the *G_Ic_* in the tests conducted at HT increases significantly with loading rate while LT makes the joints nearly insensitive to the loading rate.

**Figure 6:**
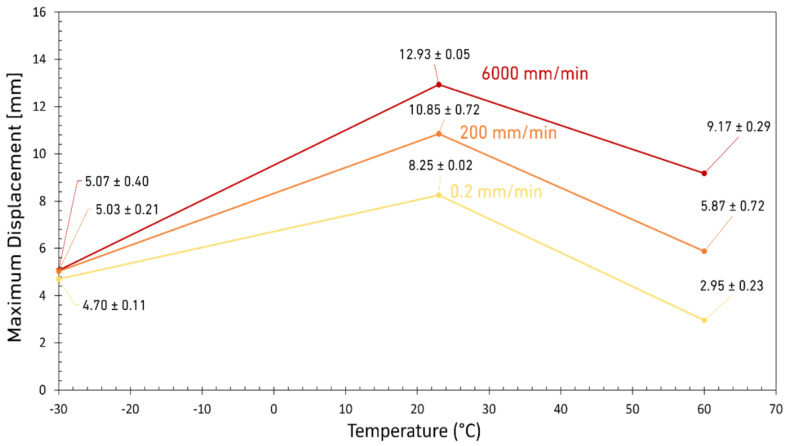
Maximum displacement as a function of temperature for three different loading rates.

**Figure 7:**
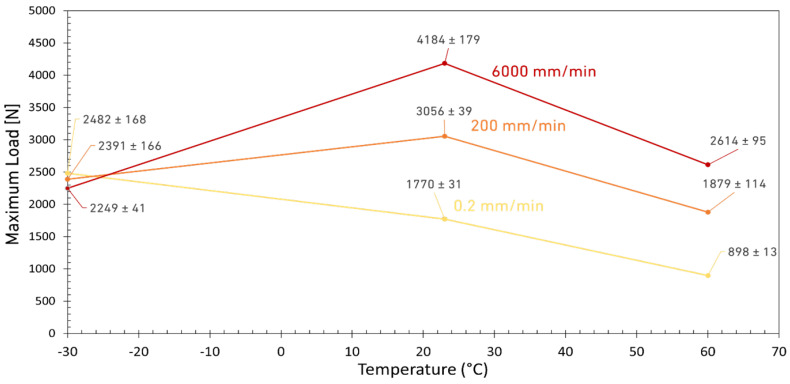
Maximum load as a function of temperature for three different loading rates.

On the other hand, the maximum strength of the tested DCB joints was less sensitive to the loading rate below *T_g_*. As shown in Figure 6, at LT, the displacement at failure is low and also insensitive to the loading rate which is due to the brittle nature of the adhesive at this temperature. However, increasing the temperature to room significantly increases the displacement at failure for all loading rates. However, as shown in Figure 6, a further increase in temperature deteriorates the adhesive properties and reduces again the displacement of the joint at failure. Based on the results shown in Figure 6, the best performance in terms of the maximum displacement at failure (which for DCB tests means a more stable crack propagation until failure) was obtained for the tests performed at RT and high loading rates. However, it should be noted that at a higher loading rate, the rate of crack growth is naturally faster due to the higher test speed. Both the displacement (Figure 6) and the strength of the joints (see Figure 7) were improved for joints tested above the *T_g_* of the adhesive. For DCBs tested below the *T_g_*, the adhesive strength is relatively higher but no significant difference was observed between the results corresponding for different loading rates. However, by increasing the temperature to room condition, the sensitivity of the adhesive to the loading rate increased which is mainly due to the more ductile and tough behavior of the adhesive being tested above its *T_g_*. According to these results, the best joint strength was obtained for high loading rate tests conducted at RT. Results shown in Figure 7 also show that any further increase in temperature deteriorates the properties of the adhesive, eventually leading lowered strength of the joints. 

**Figure 8:**
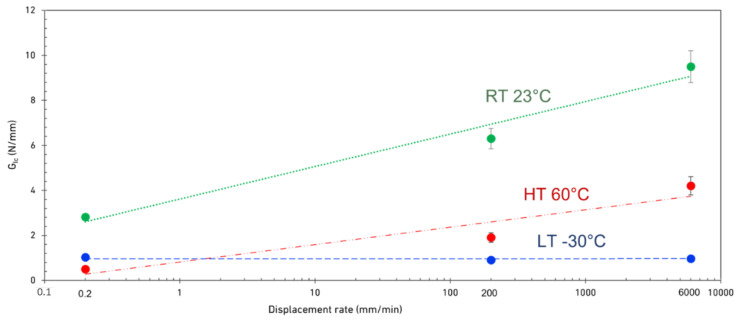
Mode I fracture energy as a function of displacement rate for different temperatures.

It should be noted that the fracture energy is effectively a product of both the load and displacement sustained by the specimen until its failure. Accordingly, to analyze the interaction of temperature and loading rate on the fracture energy, both parameters should be analyzed. As discussed above, CBBM calculates the energy of the tested joints using the compliance and the energy of bonded joints as a function of load and the loadline displacement. The results obtained are shown in Figure 8. According to these results, below the *T_g_* (at −30 °C), no significant change was observed in the mode I fracture energy as a function of loading rate. On the other hand, when tested above its *T_g_* (23 °C and 60 °C), the mode I fracture energy of the adhesive increased with loading rate where height temperature results showed the highest sensitivity to the test speed. Similar results were obtained by Machado et al. [40] and Borges et al. [25,41] for ductile epoxy adhesives. However, such a behavior was not found for the ductile polyurethane adhesive tested by Jia et al. [36], where a decrease in mode I fracture energy with increasing loading rate was reported. However, it should be noted that Jia et al. [36] tested the joints always below the *T_g_* of the adhesive. According to Figure 8, for HTs, the increase in fracture energy is relatively stronger than that identified for RT, with an increase of 8.6 times, compared to just 3.4 times between 0.2 mm/min and 6000 mm/min. This was to be expected according to the obtained tests results, as the maximum load and maximum deformation both increase strongly at HT when increasing the temperature. 

Blackman et al. [16] showed that considering the kinetic effect can change the mode I fracture energy of DCBs by 10% for joints tested at 15 m/s, while in the current study, the maximum loading rate is limited to 0.1 m/s (6000 mm/min), which is much lower than what considered in the work of Blackman et al. [16]. Accordingly, the kinetic effects were considered as negligible in the current paper.

**Figure 9:**
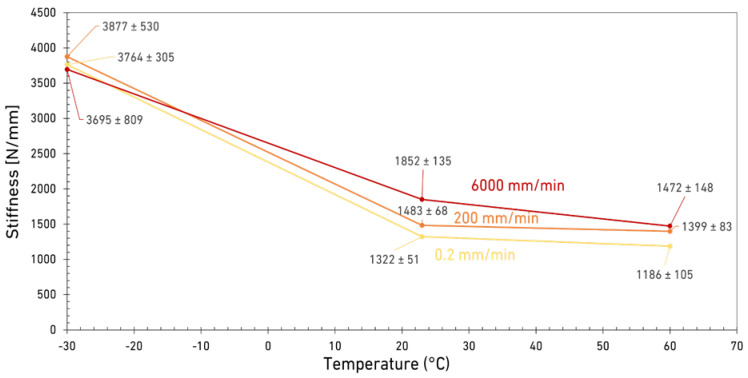
Stiffness as a function of temperature for three different loading rates.

The relative discrepancy between maximum load and displacement between RT and HT decreases when increasing the loading rate, as shown in Figure 3, Figure 4 and Figure 5. Consequently, the same statement can be made regarding Mode I fracture energy values as shown in Figure 8. These results do correlate well with the results reported by Banea et al. [29], obtained with epoxy adhesives. They showed an increase of the G_IC_ values with increasing temperature, while below the *T_g_*, there is a decrease around and above the *T_g_* [30,31,32]. For higher loading rates, the brittle behavior at LT makes the adhesive very hard to use. However, under quasi-static conditions, with its superior maximum load, stiffness, and good level of stability below the *T_g_*, the adhesive’s properties are much more interesting for engineering applications. In contrast, only above the *T_g_* do the adhesive properties appear to be excessively sensitive to the temperature. Within a well-controlled environment, always around RT, the adhesive’s fracture toughness is quite good, especially at higher loading rates. Furthermore, the same can be said for the adhesives’ strength, maximum displacement at failure, and for the stiffness (shown in Figure 9) as these values would only decrease significantly with higher temperatures. Figure 9 also shows that the stiffness of the adhesive doesn’t change significantly with temperatures above the *T_g_* while a significant change was observed between LT (below the *T_g_*) and RT (above the *T_g_*). 

**Figure 10:**
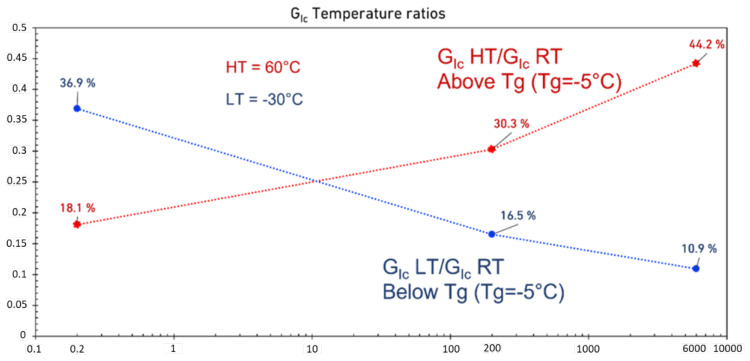
Mode I fracture energy temperature ratios between both HT and RT (red curve), and LT and RT (blue curve).

The ratio of low- and high- temperature fracture energy to the RT as a function of loading rate is shown in Figure 10. This analysis shows that increasing the loading rate has a positive impact on the HT results, while for LT, the fracture energy reduces to around 10% of the fracture energy at RT. 

Increasing the temperature decreases the strength of the adhesives and consequently, reduces their Mode I fracture energy (as shown in Figure 8). On the other hand, increasing the loading rate can compensate for this strength. Accordingly, to ensure that the fracture energy remains constant, the temperature and the loading rate should be increased at the same time. As shown in Figure 10, increasing the loading rate improves the HT fracture energy of the adhesive compared to RT results. A reduction in fracture energy was also observed by lowering the test temperature below the *T_g_* of the adhesive. This reduction is due to reductions in displacement at failure and loading. However, as shown in Figure 10, in contrast to the HT condition, not only can increasing the loading rate not compensate for the drop in strength, it reduces the fracture energy.

### Fracture Surface Analysis

**Figure 11:**
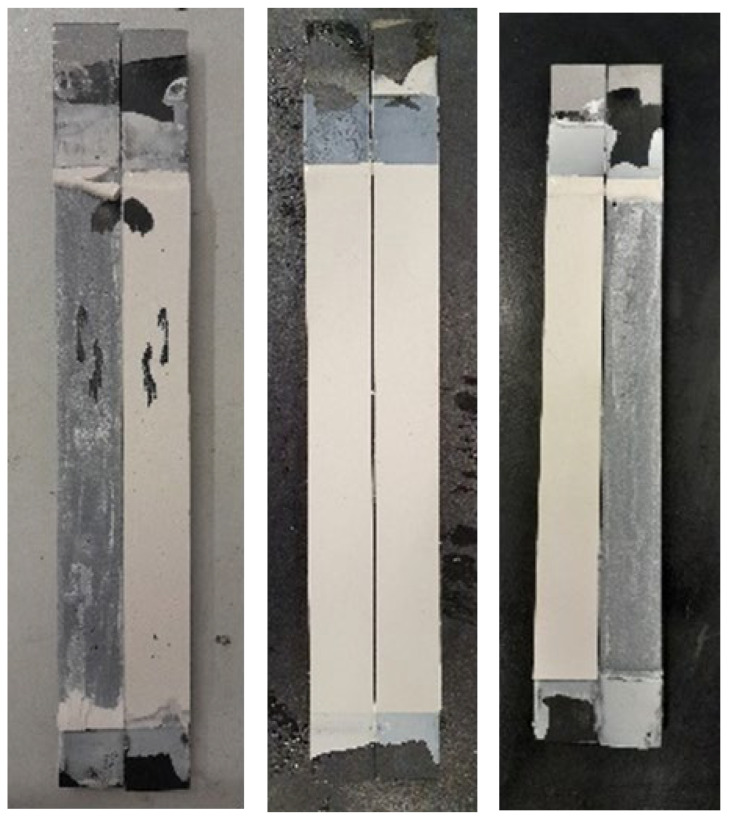
Fracture surfaces obtained under intermediate loading rate (200 mm/min) at, respectively, 23 °C (**left**), −30 °C (**middle**), and 60 °C (**right**).

**Figure 12:**
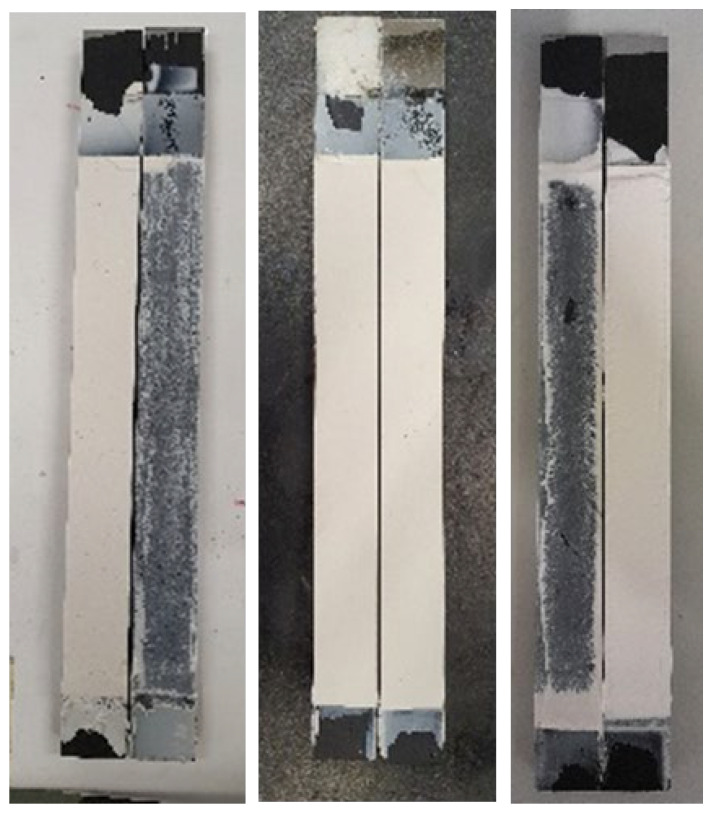
Fracture surfaces obtained under high loading rate (6000 mm/min) at, respectively, 23 °C (**left**), −30 °C (**middle**), and 60 °C (**right**).

**Figure 13:**
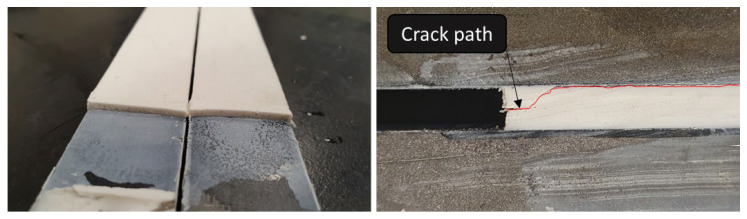
Fracture surface at the crack tip after an LT (−30 °C) mode I fracture test on the (**left**). Typical crack path around the crack tip at 23 °C or 60 °C on the (**right**).

Three different fracture mechanisms were observed. Figure 11 and Figure 12 shows the fracture surfaces of the joints tested at different conditions. The fracture surfaces at RT and HT mainly showed crack initiation at the pre-crack tip which then kinks towards one of the interfaces and propagates close to the interface, always within the adhesive layer. In quasi-static tests, however, multi-side cracking is seen. HT tests showed a smoother fracture surface, although more uneven in morphology. For LT, and since the adhesive’s behavior is brittle, cracking occurs in multiple phases, which correlates with the multiple peaks of the load-displacement curves, especially for lower speed tests. Another difference found for LT is the absence of side cracking. It is the very low ductility that prevents the crack from going to one side of the DCB joint, so the cracking follows a straight line at the mid-thickness of the adhesive layer instead of going at one of the interfaces as shown in Figure 13.

## 5. Conclusions

Polyurethane adhesives are well known for their high ductility and damping capacity. However, the high loading rate behavior of these materials is influenced by the service temperature. This work sought to determine the effect of temperature below and above the *T_g_* on the fracture behavior of a special purpose polyurethane adhesive with adapted properties based on the application. A wide range of loading rates was also considered for each test temperature.

Accordingly, the mode I fracture energy of a ductile polyurethane adhesive was experimentally analyzed under three different test speeds (0.2 mm/min, 200 mm/min and 6000 mm/min) and different temperatures (−30 °C, 23 °C and 60 °C). The main conclusions drawn from this research are the following:

1. The adhesive’s mode I fracture behavior and fracture toughness are greatly influenced by service temperature, as the influence of loading rate is quite different when the test is carried out below the *T_g_* or at temperatures above the *T_g_* of the adhesive.
2. Above the *T_g_*, the increase in loading rate increases the *G_Ic_* by a factor of 3.5, while it does not change significantly when the test is running below the *T_g_*.
3. It was found that the influence of loading rate is relatively more important for HT than for RT. Based on the results at HT and a high loading rate, the *G_Ic_* increased by a factor of 10 compared to the factor of 3.5 obtained for RT results.
4. When above the *T_g_*, the increase of temperature greatly decreases the *G_Ic_*, which is less important for higher loading rates.
5. From the stiffness analysis, it was found that the transition from LT to RT significantly decreases the bond stiffness by 60%, while further temperature increase (from RT to HT) has no significant effect on this parameter.
6. Based on the experimental results, the best performance in terms of mode I fracture energy of the adhesive was obtained for RT and at higher loading rates with the *G_Ic_* of around 9.5 N/mm.

## Figures and Tables

**Table 1:** Physical and mechanical properties of the polyurethane adhesive.

Property	Polyol	Isocyanate	Mixed
Specific gravity at 25 °C (g/cm^3^)	1.57	1.22	-
Viscosity at 25 °C (mPa.s)	7000	20	1100
Glass transition temperature (Tg) (°C)			−5
Maximum tensile strength (MPa)			3.4 ± 0.09
Maximum tensile strain (%)			33.4 ± 1.34
Young’s Modulus (MPa)			20.3 ± 1.23

**Table 2:** Summary of the test conditions.

	Temperature	LT −30 °C	RT 23 °C	HT 60 °C
Loading Rate				
Quasi-Static 0.2 mm/min		DCB, mode I fracture test	Tensile test using dogbone bulk samples and mode I fracture test using DCB specimens	DCB, mode I fracture test
Intermediate Speed 200 mm/min				
High Speed 6000 mm/min				

## Data Availability

Data is contained within the article.
